# The role of nitric oxide during embryonic epidermis development of *Xenopus laevis*

**DOI:** 10.1242/bio.023739

**Published:** 2017-05-08

**Authors:** Silvie Tomankova, Pavel Abaffy, Radek Sindelka

**Affiliations:** 1 Laboratory of Gene Expression, Institute of Biotechnology, Academy of Sciences of the Czech Republic, Průmyslová 595, Vestec 252 50, Czech Republic; 2 Charles University in Prague, Faculty of Science, Department of Genetics and Microbiology, Vinicna 5, Prague 128 43, Czech Republic

**Keywords:** Development, Epidermis, Nitric oxide, *Xenopus laevis*, Mucociliary epithelium

## Abstract

Nitric oxide (NO) is a potent radical molecule that participates in various biological processes such as vasodilation, cell proliferation, immune response and neurotransmission. NO mainly activates soluble guanylate cyclase, leading to cGMP production and activation of protein kinase G and its downstream targets. Here we report the essential role of NO during embryonic epidermis development. *Xenopus* embryonic epidermis has become a useful model reflecting human epithelial tissue composition. The developing epidermis of *Xenopus laevis* is formed from specialized ionocytes, multi-ciliated, goblet and small secretory cells. We found that NO is mainly produced in multi-ciliated cells and ionocytes. Production of NO during early developmental stages is required for formation of multi-ciliated cells, ionocytes and small secretory cells by regulation of epidermal-specific gene expression. The data from this research indicate a novel role of NO during development, which supports recent findings of NO production in human mucociliary and epithelium development.

## Abstract (precis)

**Summary:** Embryonic epidermis development is influenced by nitric oxide, where it has been linked to the development of ionocytes, multi-ciliated cells and small secretory cells.

## INTRODUCTION

*Xenopus laevis* has been recently introduced as a great model for mucociliary and epidermal research (Dubaissi and Papalopulu, 2011; Noiret et al., 2016). *Xenopus* epidermis show similarity in cellular composition with human mucus and mucociliary epithelium and also share similar genetically encoded defects especially for abnormalities during respiratory tract development (Dubaissi and Papalopulu, 2011; Walentek et al., 2014, 2015). Matured epidermis of *Xenopus* tadpoles is a bilayered epithelium (like mammalian embryonic epidermis) with the outer layer composed of specialized cell types (goblet, ion secreting, small secretory and multi-ciliated cells) and the inner layer of undifferentiated cells (Chang and Hemmati-Brivanlou, 1998).

The first differentiation of epidermal cells to precursor cells occurs during gastrulation in the inner layer (Billett and Gould, 1971). The precursors of multi-ciliated cells (MCCs) are specified by Notch-mediated lateral inhibition. Cells destined to MCC fate, express Notch ligand Delta and activate the Notch pathway in neighbouring cells. Activated Notch leads to expression of genes, which prevent transformation of these neighbouring cells to ciliated fate (Wettstein et al., 1997; Deblandre et al., 1999). Precursors of ciliated cells migrate by radial intercalation during neurulation from the inner to the outer layer in a ‘salt and pepper’ pattern (Deblandre et al., 1999; Marnellos et al., 2000; Stubbs et al., 2006). Subsequently, these cells undergo ciliogenesis at stages 22-28 (Nieuwkoop and Faber, 1994) and become mature MCCs with coordinated polarization at stage 30 (Mitchell et al., 2007). MCCs are probably responsible for the transport of extracellular fluids along the embryonic skin by cilia beating in a single polarized direction. Complex molecular regulation of ciliated epithelia development is poorly understood and its importance was shown in studies of defective ciliogenesis associated with the broad spectrum of human ciliopathies (Mall, 2008; Marshall and Kintner, 2008).

In addition to MCCs, three other cellular types can be distinguished in the outer layer of matured embryonic epidermis. Ionocytes [sometimes called intercalating non-ciliated cells (INCs)] are specified by Notch signalling during gastrulation and intercalate into the outer layer of the epidermis like MCCs, and form a scattered distribution often near or in contact with MCCs. Ionocytes are divided into two subgroups (alpha and beta subtypes) based on their differing expression levels of *foxi1e*, *ca12* and *atp6v1a.* Ionocytes are responsible for pH regulation by the activity of their membrane pumps and channels and they also play a crucial role in the development of a functional mucociliary epithelium. Depletion of ionocytes leads to defects in MCCs (Stubbs et al., 2006; Dubaissi and Papalopulu, 2011; Quigley et al., 2011). Recently, another cell type within the outer layer of the epidermis, the small secretory cells (SSCs), has been characterized. SSCs contain apically localized large vesicles containing serotonin, which is a monoamine derived from tryptophan modification. Development of SSCs is regulated by the activity of the transcription factor Foxa1. Expression of *foxa1* first appears in the inner layer during early neurulation (Dubaissi et al., 2014). SSCs intercalate into the outer layer of the epidermis during stages 27-30, later than that of MCCs and ionocytes. Matured SSCs synthesize and secrete serotonin and additional substances on the surface of the embryo to provide it some anti-bacterial function. SSCs regulate motility and beating frequency of MCCs through serotonin receptor Htr3 at the later stages of embryonic development (Dubaissi et al., 2014; Walentek et al., 2014). Goblet cells are the fourth epidermal cell type. In contrast to the other cell types, goblet cells do not intercalate from the inner epidermal layer, but instead develop and form most of the outer layer. Goblet cells are known to secrete a lectin Xeel and probably have a protective function in the embryonic epidermis (Billett and Gould, 1971; Nagata et al., 2003).

It is well known that matured epidermis is susceptible to many chemical and physical factors. One of the main factors influencing skin is UV light, which causes production of reactive oxygen species (ROS) and reactive nitrogen species (RNS) within the epidermis (Peus et al., 1998; Fisher et al., 2002; Chang et al., 2003; Ichihashi et al., 2003). Although there is plenty of information about the negative effect of ROS and RNS on matured epidermis, almost nothing is known about their positive role during embryonic epidermis development. A very interesting molecule from the RNS group is nitric oxide (NO), which is a small signalling molecule involved in a wide range of biological processes (both physiological and pathological) like vasodilation, neurotransmission and apoptosis (Palmer et al., 1987; Albina et al., 1993; West et al., 2002). It is generated by three nitric oxide synthases (NOS): constitutively active neuronal, nNOS/NOS1; endothelial, eNOS/NOS3; and indubible, iNOS/NOS2 (Bredt et al., 1991; Knowles and Moncada, 1994). NO activates a soluble isoform of guanylyl cyclase (sGC) that induces the production of cGMP followed by the activation of protein kinase G (PRKG) regulating transcription and translation of downstream target genes (Arnold et al., 1977; Francis et al., 2010), and, alternatively, acts independently on the cGMP pathway (Cui et al., 2005; Oberprieler et al., 2007). NO production has been detected in the human skin (Wang et al., 1996; Shimizu et al., 1997; Chang et al., 2003), but its function is unclear.

NO is crucial during embryogenesis, especially during craniofacial development (Gouge et al., 1998; Jacox et al., 2014). NO was found to be highly produced in embryonic epithelia of various animal models (Brunelli et al., 2005; Alpert et al., 2007; Wildling and Kerschbaum, 2007; Jacox et al., 2014). Furthermore, its production and function were studied in the human pulmonary epithelium, ciliary motility and ciliary beating frequency (Jain et al., 1993; Jiao et al., 2010). Neuronal version of NOS was discovered in human cilia airway epithelium localized at the proximal portion of cilia and it is responsible for rapid and localized NO production (Jackson et al., 2015).

Here we expand our knowledge about the biological function of NO during embryonic epidermis formation in *Xenopus laevis*. We found out that NO regulates expression of genes which are important for epidermis development. Moreover, we discovered that the production of NO is cell specific and its inhibition leads to defects in MCCs, ionocytes and SSCs development. Furthermore, we showed correlation between NO signalling and Notch pathway. In summary, we propose a novel function of NO during development, regulation of epidermal formation, which is probably necessary for embryonic epidermis development and can be crucial for human mucus and mucociliary epithelium function.

## RESULTS

### Identification of epidermal cell types producing NO

**Fig. 1.**
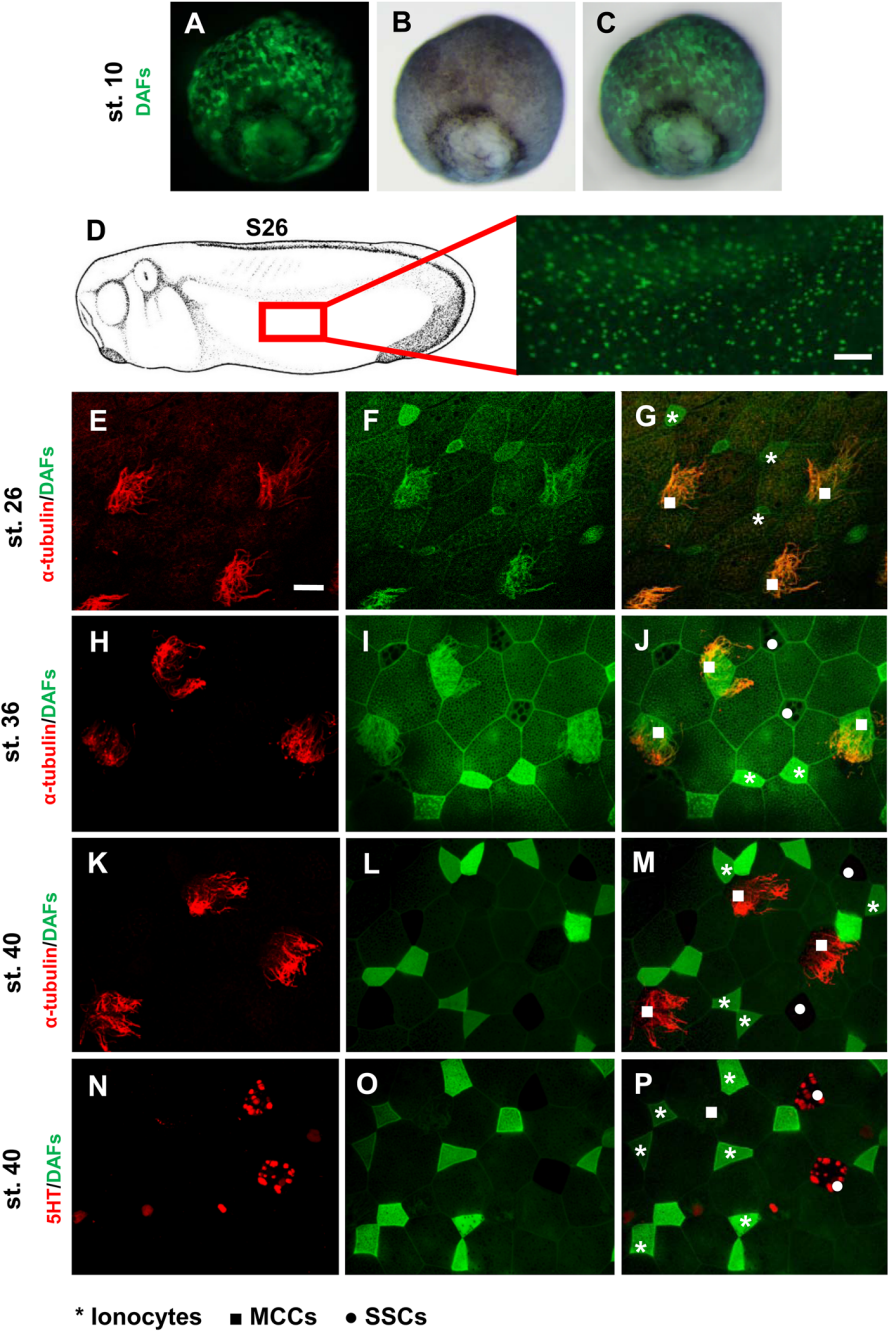
**Production of NO in embryonic epidermis.** (A-C) NO staining by DAFs was made in early developmental stages (stage 10). (D) NO is produced in certain cells in a ‘salt and pepper’ manner at stage 26 (*n*=20). Scale bar: 200 µm. Embryos were labelled with anti-alpha tubulin (MCCs) at stage 26, 36 and 40 (E,H,K) and with anti 5-HT (vesicles in SSCs) (N). Double staining with DAFs (NO) and anti-alpha tubulin (MCCs) at stage 26, 36 and 40 (G,J,M) or DAFs and anti-5HT (SSCs) at stage 40 (P) was made to determine the cell type where is NO produced. NO is produced in MCCs at stage 26 and 36 (F,G,I,J) and in ionocytes at stage 26, 36 and 40 (F,G,I,J,L,M,O,P). The production of NO was not detect in SSCs (I,J,L,M,O,P) and in MCCs at stage 40 (L,M). Ionocytes form couples at stage 40 (L,M,O,P). At least five embryos were used for each condition. Scale bar: 20 µm in E, magnification 40×.

**Fig. 2.**
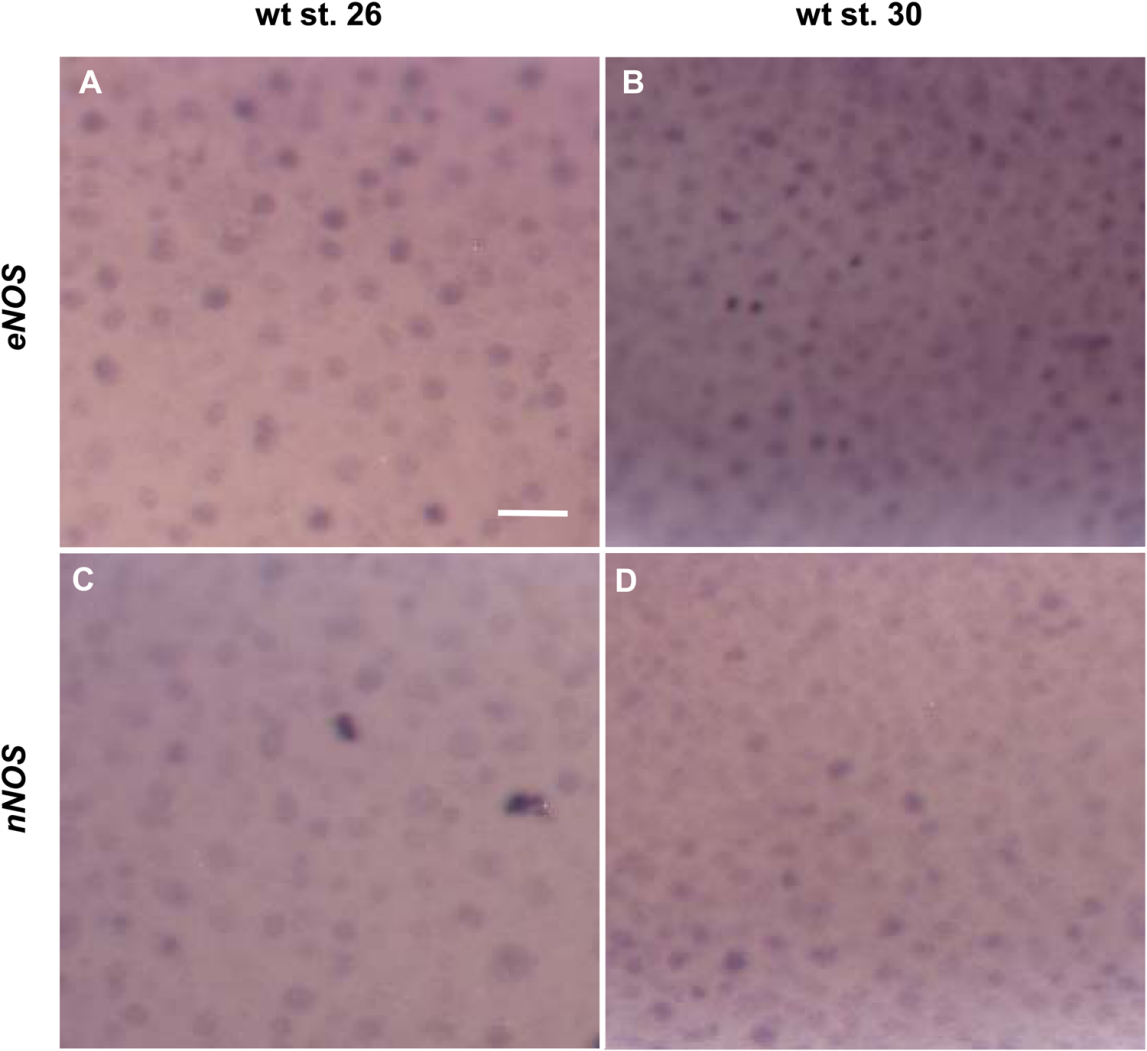
**Occurrence of NOS in embryonic epidermis.** Embryos were hybridised with probes against *eNOS* (A,B) and *nNOS* (C,D) at stage 26 (A,C) and 30 (B,D). Positive cells on *eNOS*, *nNOS* are scattered through embryonic epidermis (magnification 11,25×, scale bar: 50 µm). At least five embryos were used for each condition.

NO production and *nNOS* expression were previously identified in the most outer layer of the developing epidermis in the sagittal sections at stage 20 (Jacox et al., 2014). Our goal here is to show detailed analysis of NO and *nNOS* at different embryotic stages. We performed NO staining at stages 10, 26, 36 and 40 and *in situ* hybridization using probes against *eNOS* and *nNOS* at stages 26 and 30 (Figs 1 and 2). Production of NO is apparent in early developmental stages (stage 10) in *Xenopus* embryos (Fig. 1A-C). NO staining revealed the ‘salt and pepper’ pattern at stage 26, characteristic for specific epidermal cell-type activity (Fig. 1D). We combined NO staining with immunohistochemistry using antibody markers for MCCs (α-tubulin) and SSCs (Serotonin) at stages 26 (Fig. 1E-G), 36 (Fig. 1H-I) and 40 (Fig. 1K-P) to identify the cell type producing NO. Our results showed that the NO is produced in ionocytes throughout the studied early embryonic development (Fig. 1G,J,M,P) and in MCCs at stages 26 and 36 (Fig. 1G,J). NO was not detected in MCCs at stage 40 (Fig. 1M,P). Production of NO was not detected in either SSCs nor goblet cells (Fig. 1P). Interestingly, expression of *eNOS* (Fig. 2A,B) and *nNOS* (Fig. 2C,D) correlates with NO production. Both are expressed in the same ‘salt and pepper’ pattern at stages 26 and 30 (Fig. 2).

### NO regulates expression of genes, which are necessary for epidermis development and function

It is known that NO regulates gene expression (Bogdan, 2001; Pfeilschifter et al., 2001). We performed transcriptome comparison between embryos with inhibited NO production and sibling controls. RNA-Seq data contained expression of 28,399 genes at stage 26, 1702 of them showed dependence NO (a statistically significant adjusted *P* value lower than 0.1 and significant difference higher than 2×), 728 were upregulated, and 974 were downregulated (Fig. S1). Table 1 summarises the top genes which are regulated by NO. Interestingly, lots of downregulated genes were related to epidermis development and function (especially collagens and keratins). We selected 37 candidate genes from transcriptome analysis for validation and performed RT-qPCR (Fig. S1C). Comparison between the results from RNA-Seq and RT-qPCR showed similarity (R^2^=0.6316).

**Table 1.**
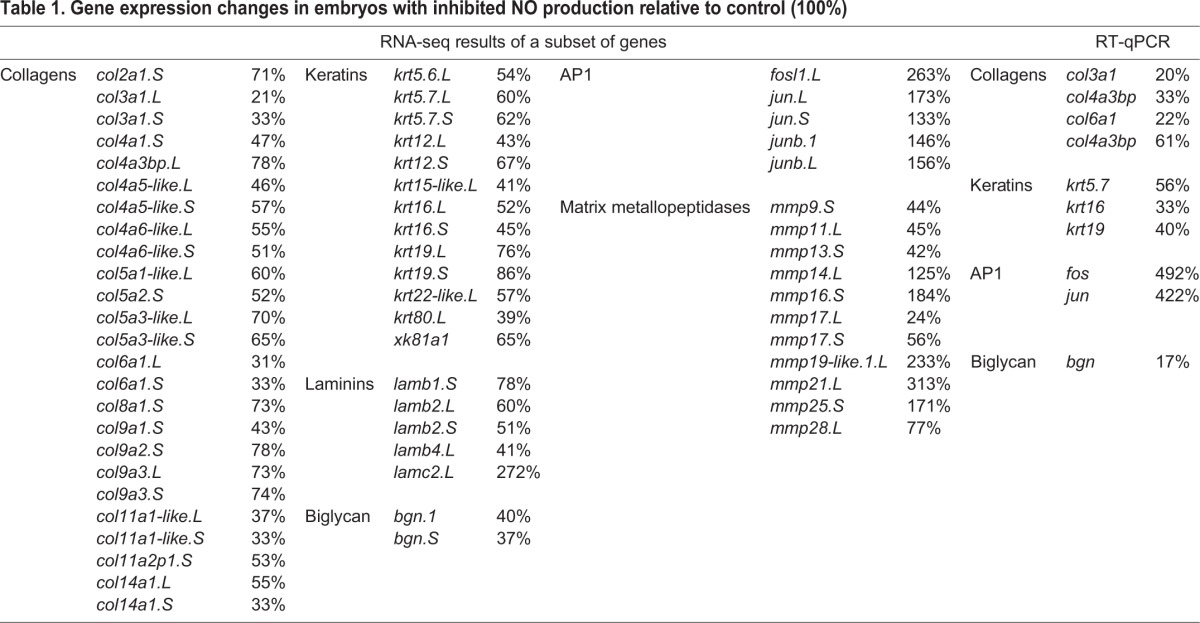
**Gene expression changes in embryos with inhibited NO production relative to control (100%)**

To test downregulation of collagens at protein levels we performed Masson’s trichrome staining using control and NO-inhibited embryos at stage 26. Our results demonstrate that inhibition of NO lead to decrease in the amount of collagens protein more than 30% (Fig. S2).

### Inhibition of NO causes decrease in number of specific epidermal cell types

**Fig. 3.**
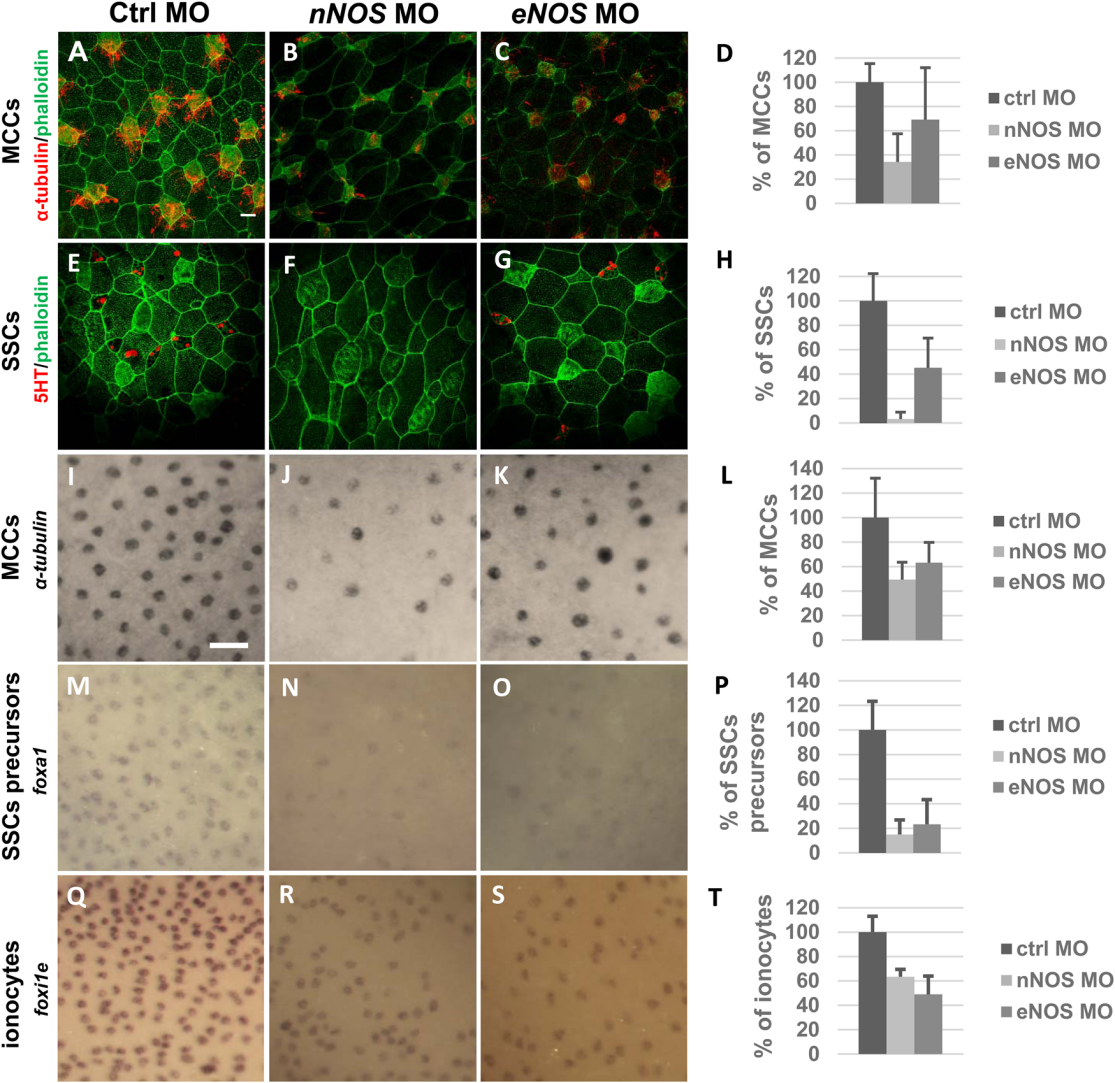
**Inhibition of NO causes defects in ionocytes, multi-ciliated and small secretory cells.** Embryos were injected by ctrl MO 34 ng (A,E,I,M,Q), *nNOS* MO 17 ng (B,F,J,N,R) and *eNOS* MO 34 ng (C,G,K,O,S) at stage 1 and fixed in 4% PFA at stage 26 (I-S), 30 (A-C) and 36 (E-G). MCCs were labelled with anti-alpha tubulin (A-C), vesicles in SSCs were labelled with anti-5HT (E-G) and membranes with antibody against phalloidin (A-G) and imaged (apical surface of the embryonic epidermis) by confocal microscopy (magnification 40×, scale bar: 20 µm) (A-G). MCCs, SSCs and ionocytes were marked by *alpha-tubulin* (I-K), *foxa1* (M-O) and *foxi1e* probe (Q-S) and imaged by macroscope (magnification 11,25×, scale bar: 50 µm) (I-S). Embryos showing frequency of individual cell types after applying ctrl (A,E,I,M,Q), *nNOS* (B,F,J,N,R) and *eNOS* (C,G,K,O,S) MO. Injection of *nNOS* MOS (B,F,J,N,R) and *eNOS* MO (C,G,K,O,S) cause reduction in number of MCCs (D), SSCs (H) and reduced expression of scattered cell markers *alpha-tubulin* (L), *foxa1* (P) and *foxi1e* (T). Quantification of incidence of MCCs (D,L), SSCs (H,P) and ionocytes (T), error bars indicate +s.d., the *P*<0.005 except ctrl×*eNOS* (D) where *P*>0.005. At least five embryos were used for each condition (one-way ANOVA, Tukey's multiple comparisons test).

Production of NO in the epidermis led us to hypothesize that NO is required for epidermis development and cell specific formation. We performed loss-of-function experiments using chemical inhibitors and morpholinos (*nNOS* MO and *eNOS* MO). We continued with immunofluorescence staining at stages 30 and 36 using antibody markers for MCCs (α-tubulin) and SSCs (serotonin) (Fig. 3A-H) and *in situ* hybridization at stage 26 using probes against *foxi1e* (marker for ionocytes), *foxa1* (marker for SSCs), *α-tubulin* (marker for MCCs) (Fig. 3I-T) and *otogelin* (marker for goblet cells) at stage 26 (Fig. S3). We compared the numbers of individual epidermal cell types between treated and control embryos. We observed a significant reduction in the number of MCCs, SSCs and ionocytes in embryos with inhibited NO production (Fig. 3D,H,L,P,T). Immunofluorescence staining showed a decrease in SSCs by 97% (*nNOS* MO) (Fig. 3F,H) and by 55% (*eNOS* MO) (Fig. 3G,H) at stage 36. MCCs decreased by 66% (*nNOS* MO) (Fig. 3B,D) and by 31% (*eNOS* MO) (Fig. 3C,D) at stage 30 (counted every cell with at least one cilia). Further NO inhibition lead to dramatic decrease of number of cilias and their length (Fig. S4) and phenotypic changes at the level of basal bodies (accumulation of gamma tubulin in the central region of MCCs in embryos with inhibited NO production compared with ctrl MO embryos) (Fig. S5). At least 50% of treated embryos showed this phenotype. Taken together, immunofluorescence staining indicates abnormal ciliogenesis in embryos with inhibited NO production.

*In situ* hybridization for MCCs marker showed decrease by 50% (*nNOS* MO) (Fig. 3J,L) and by 36% (*eNOS* MO) at stage 26. SSCs precursors decreased by 85% (*nNOS* MO) (Fig. 3N,P) and by 77% (*eNOS* MO) (Fig. 3O,P) at stage 26. Furthermore, we also observed a decrease of ionocytes by 37% (*nNOS* MO) (Fig. 3R,T) and by 51% (*eNOS* MO) (Fig. 3S,T) at stage 26. These results indicates that NO is required for the formation of MCCs, SSCs and ionocytes.

Changes in epidermis development were supported also by gene expression analysis using samples of embryonic epidermis at stage 26. Decrease of other epidermal markers for MCCs (*tuba1a*, *foxj1*), SSCs (*foxa1a*) and ionocytes (*foxi1e*, *atp6v1a*) was found in embryos with inhibited NO and, in contrast, marker of goblet cells (*itln2*) showed increase (Fig. S6).

**Fig. 4.**
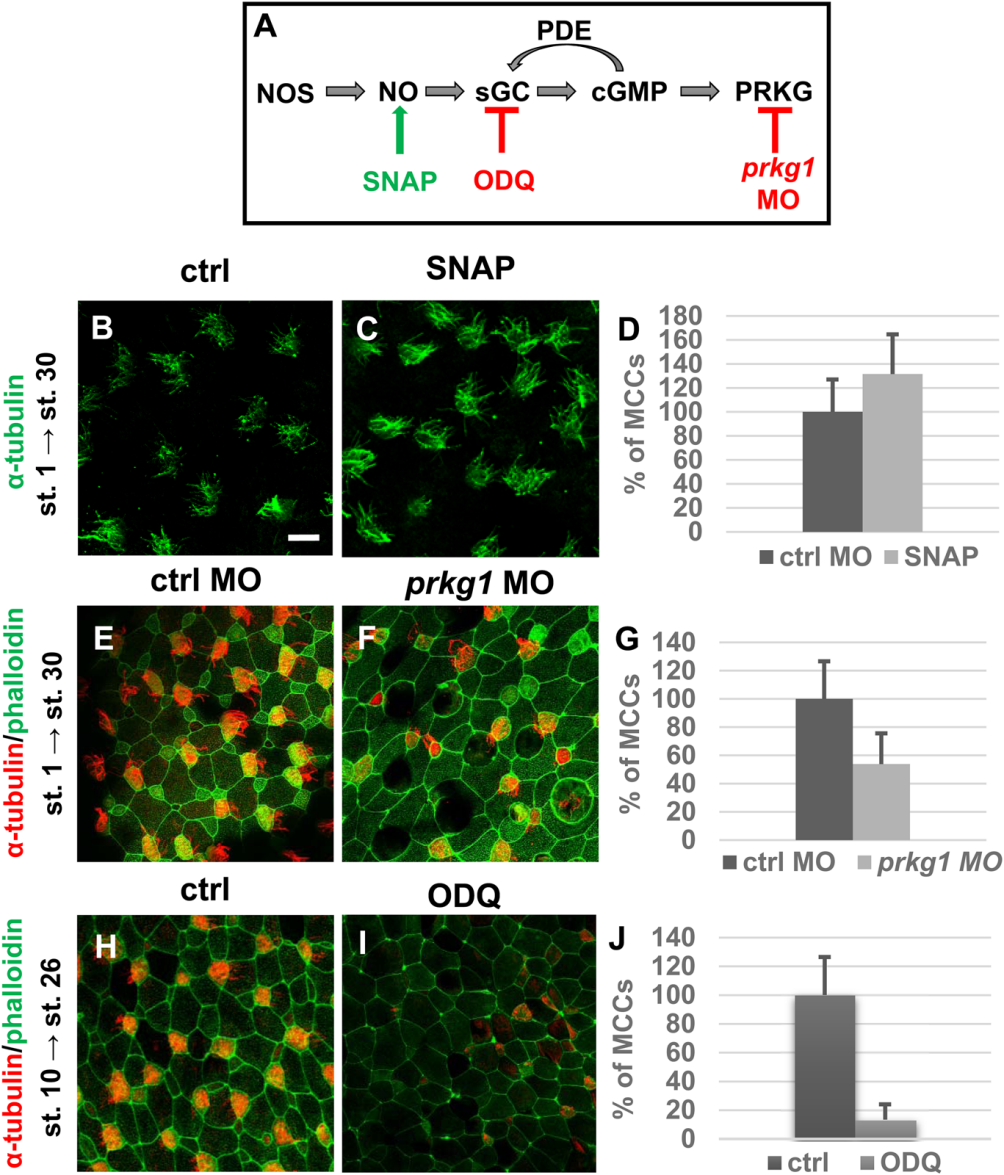
**NO/cGMP signalling pathway and its effect on embryonic epidermis.** (A) The NO pathway with used inhibitors in this experiment. (B,C,E,F) Embryos were injected with DMSO 1 nl 100 mM (B), SNAP 1 nl 100 mM (C), ctrl MO 51 ng (E) and *prkg1* MO 51 ng (F) at stage 1 and fixed in 4% PFA at stage 30. Embryos were stained with an antibody against α-tubulin (MCCs) and phalloidin (membranes) and imaged (apical surface of the embryonic epidermis) by confocal microscopy (magnification 40×, scale bar: 20 µm). Injection of SNAP increased number of MCCs and *prkg1* MO reduced number of MCCs in embryonic epidermis. (D,G) Quantification of incidence of MCCs, error bars indicate +s.d., *P*<0.04 (D) and *P*<0.002 (G). At least five embryos were used for each condition (Student's *t*-test). The vitelline membrane was removed from embryos at stage 8 to 9. Treatment by ODQ were performed at stage 10 to 26 and embryos – affected (I) and control (H) were collected at stage 26. Embryos were stained with an antibody against α-tubulin (MCCs) and phalloidin (membranes) and imaged (apical surface of the embryonic epidermis) by confocal microscopy (magnification 40×, scale bar: 20 µm). Treatment by ODQ reduced number of ciliated cells in embryos at stage 26. (J) Quantification of incidence of MCCs, error bars indicate +s.d., *P*<1×10^−8^. At least five embryos were used for each condition (Student's *t*-test).

The NO donor SNAP (S-Nitroso-N-Acetyl-D,L-Penicillamine) caused opposite effects to the loss-of-function experiments – we observed significant increase by 132% in the number of MCCs at stage 30 (Fig. 4B-D). Results from both experiments indicate the necessity of NO for the development of specific epidermal cell types.

### NO acts during epidermis formation through classical sGC-cGMP-PRKG pathway

One of the most studied mechanisms of NO activity is its activation of sGC followed by the production of cGMP and the subsequent activation of PRKG (Arnold et al., 1977; Francis et al., 2010) (Fig. 4A). We used loss of function of Prkg1 (by prkg1 MO at stage 1, Fig. 4E-G) and specific chemical inhibitor against sGC (ODQ at stage 10, Fig. 4H-J) to determine whether NO regulates epidermis development through the sCG-cGMP-PRKG pathway. Our results showed a significant decrease in the number of MCCs (46% decrease) in the embryos with inhibited prkg1 (Fig. 4F,G) and 87% decrease of MCCs using chemical inhibitor (Fig. 4I,J). This supports mechanism of NO activity through classical sGC-cGMP-PRKG pathway during epidermis development.

### NO is required during early embryonic stages for epidermis development

**Fig. 5.**
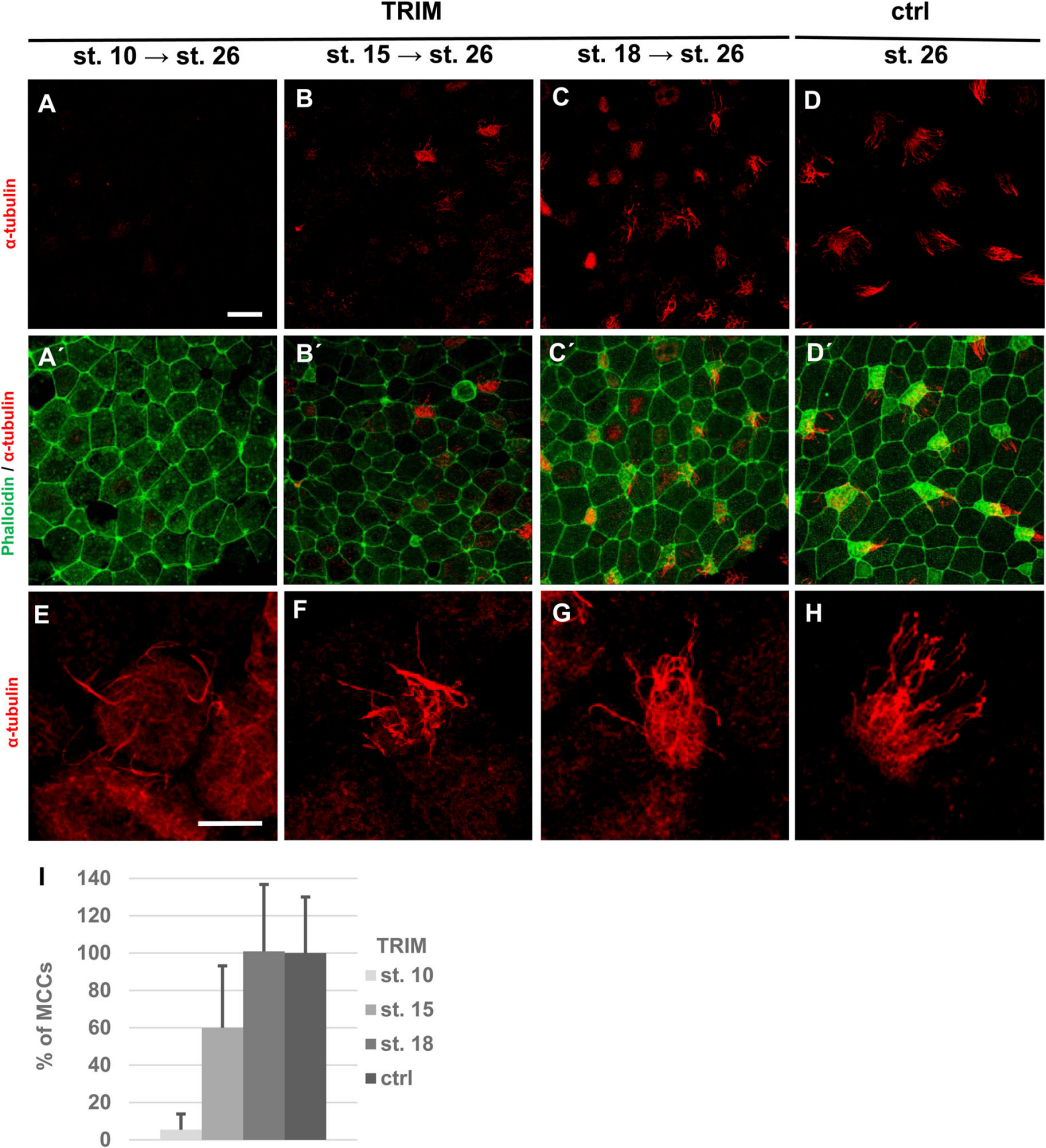
**Determination of developmental stages for necessity of NO.** The vitelline membrane was removed from embryos at stage 8 to 9. Treatment by TRIM were performed at stage 10 (A,A′,E), 15 (B,B′,F) and 18 (C,C′,G) and all embryos, treated and control (D,D′,H), were collected at stage 26. Embryos were stained with an antibody against α-tubulin (MCCs) and phalloidin (membranes) and imaged (apical surface of the embryonic epidermis) by confocal microscopy (magnification 40×, scale bar: 20 µm). Treatment with TRIM reduced number of MCCs in embryos treated at stage 10 and 15 (A-B). (I) Quantification of incidence of MCCs error bars indicate +s.d., *P*<0.03 (ctrl×st. 10, ctrl×st. 15), no statistically significant difference between ctrl embryos and embryos treated by TRIM at stage 18 (C-D). At least five embryos were used for each condition (one-way ANOVA, Tukey's multiple comparisons test).

Epidermal precursor cells are formed during gastrulation. We tested temporal requirement of NO using the specific chemical inhibitor of NOS called TRIM. We removed manually vitelline membrane and treated the embryos with TRIM from stages 10 (early gastrulation), 15 (early neurulation) and 18 (neurulation) until stage 26. Treated embryos were then collected and immunofluorescence staining using antibody markers for MCCs (α-tubulin) was performed (Fig. 5). We found almost complete loss of MCCs in embryos with inhibited NO production by TRIM from stage 10 (95% decrease) (Fig. 5A,A′,E,I). Embryos treated at stage 15 showed 40% decrease in MCCs (Fig. 5B,B′,F,I) and no difference was observed in embryos treated after stage 18 (Fig. 5C,C′,D,D′,G,H,I).

Our data indicates that NO is important for MCCs formation during the early stages of ciliogenesis and its importance disappears after neurulation.

### Notch pathway is connected with NO signalling

**Fig. 6.**
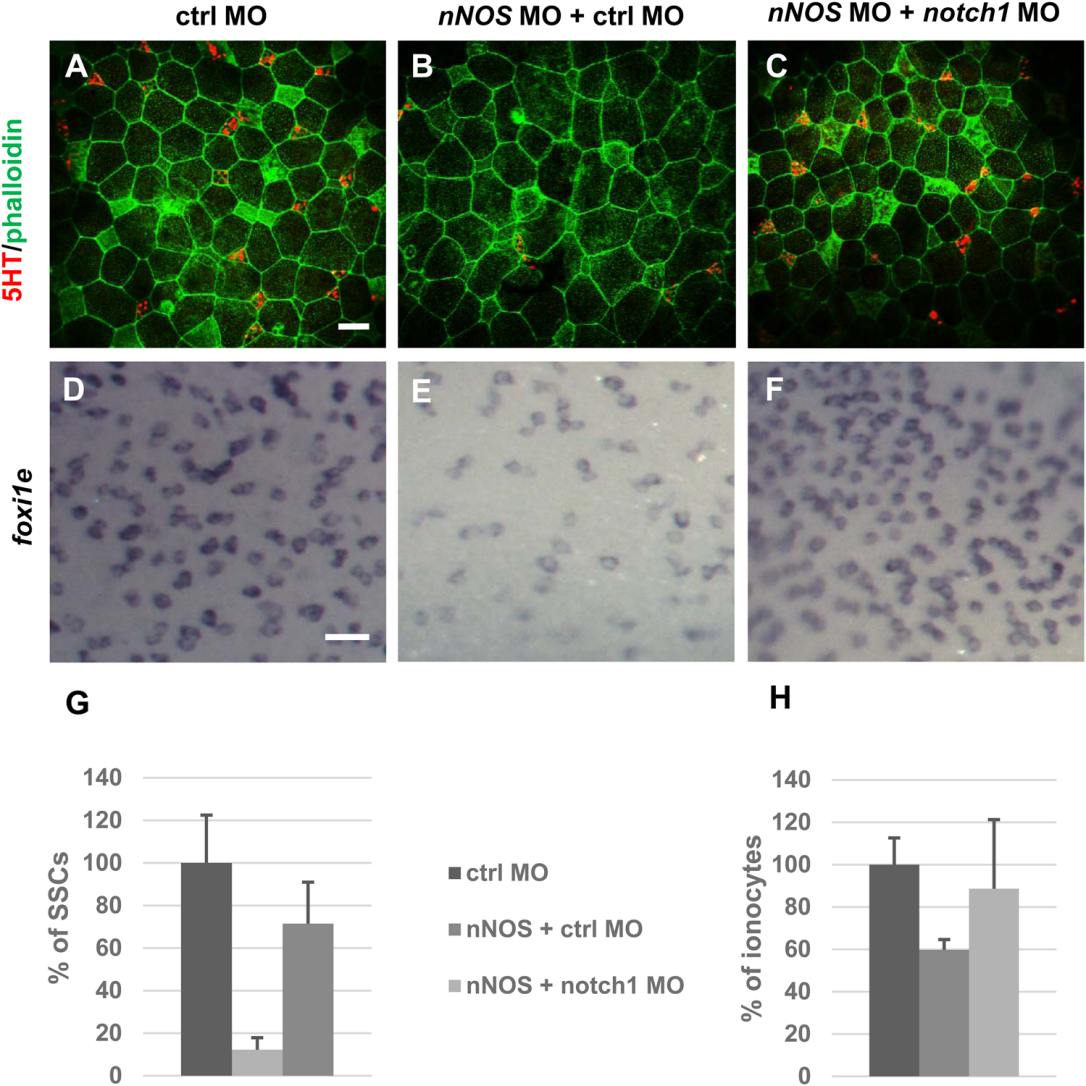
**Partial restoration of the quantity of ionocytes and SSCs by *notch1* MO.** Embryos were injected by ctrl MO 34 ng (A,D), *nNOS* MO 17 ng+ctrl MO 17 ng (B,E) and *nNOS* MO+*notch1* MO (1:1, 17 ng+17 ng) (C,F) at stage 1 and fixed in 4% PFA at stage 30 (A,B,C) and 40 (D,E,F). (A-C) SSCs were labelled by anti-5HT and membranes by antibody against phalloidin and imaged (apical surface of the embryonic epidermis) by confocal microscopy (magnification 40×, scale bar: 20 µm). (D-F) Ionocytes were marked by *foxi1e* probe (magnification 11,25×, scale bar: 50 µm). Injection of *nNOS* MOS (B,E) caused reduction in number of SSCs (B,G) and ionocytes (E,H). Combination of MO cause partial restoration of the quantity of ionocytes and SSCs (C,F,G,H). (G,H) Quantification of incidence of SSCs (G) and ionocytes (H), error bars indicate +s.d., *P*<0.0002 (ctrl×*nNOS*+ctrl MO), *P*<0.13 (ctrl×*nNOS*+*notch1* MO), *P*<0.005 (nNOS+ctrl×*nNOS*+*notch1* MO) (G), *P*<0.2 (ctrl×*nNOS*+ctrl MO), *P*<0.7 (ctrl×*nNOS*+*notch1* MO) *P*<0.35 (nNOS+ctrl×*nNOS*+*notch1* MO) (H). At least five embryos were used for each condition (one-way ANOVA, Tukey's multiple comparisons test).

Notch pathway is essential for formation of precursors of the individual epidermal cell types. Exogenous activation of Notch pathway causes reduction of MCCs, SSCs and ionocytes. Whereas Notch pathway inhibition enhances ciliogenesis (shown in *Xenopus* epidermis and human airway epithelium) leading to the multiplication of MCCs precursors and ionocytes (Deblandre et al., 1999; Stubbs et al., 2006; Hayes et al., 2007; Tsao et al., 2009; Marcet et al., 2011; Quigley et al., 2011). Our previous results showing reduction of specific epidermal cell types by NO inhibition indicates possible connection of NO production with Notch signalling. We performed synergy experiments using inhibition of Notch pathway (by *notch1* MO) and inhibition of NO production. Our hypothesis was that Notch inhibition leading to increase of MCCs may reverse the negative effect caused by NO inhibition. We combined *nNOS* MO with *notch1* MO (1:1) and compared it with *nNOS* inhibited embryos and control MO. We used immunohistochemistry staining with antibody markers 5HT/Serotonin for SSCs at stage 30 (Fig. 6A-C) and *in situ* hybridization using probe against *foxi1e* (marker for ionocytes) at stage 40 (Fig. 6D-F). Our results showed significant restoration of the quantity of ionocytes (11% decrease) and SCCs (29% decrease) compared to controls (Fig. 6A,D,G,H). In the same experiment we measured 40% reduction of ionocytes (Fig. 6E,H) and 88% reduction in SSCs (Fig. 6B,G) in *nNOS* loss-of-function embryos. MCCs staining has not shown reproducible significant reversal effect. We conclude from these results that there is connection between Notch pathway and NO production, but more experiments would be needed to reveal the mechanism.

## DISCUSSION

The NO molecule is a potent biological factor regulating various processes from blood pressure control, inflammation and neurotransmission to gene expression stimulation. NO is usually highly produced in blood vessels and wounded tissues and recently it has been detected in developing epidermis of *Xenopus* (Brunelli et al., 2005; Alpert et al., 2007; Wildling and Kerschbaum, 2007; Jacox et al., 2014) and human airways epithelia (Jiao et al., 2010; Jackson et al., 2015). Surprisingly NO is detected at developmental stages when inflammatory and blood systems are missing. Epidermal production of NO therefore indicates a novel role in early development of this protective layer of embryonic body.

Here, we performed extensive characterization of epidermal NO production and its role during development. We found that NO is produced in ionocytes throughout the early development and in multi-ciliated cells (MCCs) during ciliogenesis. Interestingly, NO was not detected in small secretory cells (SCCs). Our data correlate nicely with NO production in human airways epithelia, where NO is strongly produced in MCCs cells (Jackson et al., 2015). Potential importance of NO production in the developing epidermis is further supported by expression of both constitutively active enzymes producing NO [endothelial (eNOS)- and neuronal (nNOS)-NO synthases]. Both enzymes are expressed at the mRNA and protein levels during epidermis formation and create the typical ‘salt and pepper’ pattern similar to the presence of MCC, ionocyte and SSC cells in the developing epidermis. Immunohistochemistry using antibody against nNOS showed the same pattern (data not shown). The third NOS called inducible (iNOS) is expressed weakly during early embryogenesis and we were not able to detect its expression in developing epidermis (data not shown). Expression of iNOS is usually activated following injury as inflammatory response and leads to production of high and toxic levels of NO (Xu et al., 2001). We hypothesize that the physiological level of NO production is needed for development of epidermis and its function, and that eNOS and nNOS are required for keeping correct NO levels in cells.

Production of NO in the early ionocytes and MCCs indicates the potential role during epidermis development. We performed loss-of-function experiments using gene-specific knock downs of *nNOS* and *eNOS*. In both cases, this leads to chronic reduction of NO production and strong phenotypic changes in epidermis. Reduction of NO production had a severe effect on development of MCCs, SSCs and ionocytes. The main difference was significant reduction of the numbers of these specialized cells. Connection between development of MCCs, SSCs, and ionocytes was shown recently (Dubaissi and Papalopulu, 2011; Dubaissi et al., 2014) and our results support the theory of influence of SSCs and ionocytes on MCCs development. Reduction of cell numbers was higher for *nNOS* loss-of-function compared to *eNOS*, which indicates a dominant role of nNOS during epidermis development. Preliminary experiments using a combination of *nNOS* and *eNOS* loss of function showed synergistic effect in combined experiments (data not shown). Detailed analysis of MCCs in embryos with inhibited NO production revealed also phenotypic changes of cilia's length and reduced number of cilia per MCC (Fig. S3), together with abnormal basal bodies accumulation in the central region of MCCs indicating ciliogenesis defects.

Temporal requirement of NO production during epidermis development was studied by acute NO production inhibition using a chemical inhibitor of NOS called 1-[2-(trifluoromethyl)phenyl]imidazol (TRIM). This inhibitor specifically inhibits the function of nNOS and iNOS and, with lower efficiency, also eNOS (Handy et al., 1996). We found that NO production is required at the very early stages of epidermis specification following gastrulation. Treatment of embryos at the gastrula stage with NOS inhibitor resulted in almost total absence of MCCs development. Inhibition of NO production during later developmental stages showed lower or no effect on MCCs and ionocytes development.

A probable mechanism of NO activity during epidermis development is regulation of gene expression, which is the usual outcome of the physiological production of NO (Pilz et al., 1995; Tapia-Limonchi et al., 2016). We performed transcriptome analysis in embryos with chronic reduction of NO production (Fig. S1). Although we compared transcriptome changes of the whole embryos, a lot of genes with highest altered expression were related to epidermis development and function. We found a reduction of the mRNA levels for members of laminin, keratin, collagen or matrix metalloprotease families, which are considered to be essential for epidermis function. In addition to changes in gene expression, we showed strong reduction in collagen layer in developing epidermis after NO inhibition, which has potential impact on skin function as a barrier (Fig. S2).

Early production of NO in developing epidermis indicates a potential connection with a Notch signalling cascade, which is well known to have a key role for epidermis development. Our preliminary results showed correlation of NO production with Notch signalling, but it is not yet possible to deduce the mechanism of their activities and more thorough studies are required.

The present study highlights the importance of NO during embryonic epidermis formation and its potential for the study of NO production and function in human lung epithelium and related defects.

## MATERIAL AND METHODS

### Embryos manipulation

*Xenopus laevis* eggs were fertilized *in vitro* and cultured in 0.1×Modified Barth's Saline (MBS: 8.8 mM NaCl, 0.1 mM KCl, 0.5 mM HEPES pH 7.8, 0.07 mM CaCl_2_, 0.1 mM MgSO_4_, 0.25 mM NaHCO_3_)+gentamicin (20 µg/ml) until stage 26-40. Embryos were staged according to the designations established by Nieuwkoop and Faber (1994). Experiments complied with all relevant institutional and national animal welfare laws, guidelines and policies, as should the care and use of experimental animals.

### Quantitative real-time PCR

Total RNA from *X. laevis* embryos was extracted using TRI Reagent^®^ (Sigma-Aldrich, T9424) following their standard protocol. cDNA was then synthesized from 100 ng of total RNA by Superscript III reverse transcriptase (Invitrogen™, 18080093) with thermocycling condition: 75°C for 5 min, 25°C for 20 s, 4°C for 2 min, 25°C for 5 min, 50°C for 60 min, 55°C for 15 min, 75°C for 15 min. qPCR analysis was performed on the C1000™ Thermal Cycler CFX384™ Real-time system (Bio-Rad) with iQ™ SYBR Green Supermix (Bio-Rad, 1708880) using thermocycling condition: initial denaturation at 95°C for 3 min, 40 cycles of denaturation at 95°C for 15 s, annealing at 60°C for 20 s and elongation at 72°C for 20 s followed by melting curve analysis. Biological triplicate reactions were performed for each condition including negative controls and gDNA contamination controls. Primer sequences are available on request.

### Morpholino oligonucleotides

All morpholinos (MO) were purchased from Gene Tools, LLC (Philomath, OR, USA). Standard control MO designed by Gene Tools was used in control injections. Sequence of the *nNOS* spliced morpholino was obtained from Jacox et al. (2014) (5′-TGGCTAAAAGAACACAGGACATCAA-3′), while the sequence of *notch1* MO was obtained from www.xenbase.org (5′-GCACAGCCAGCCCTATCCGATCCAT-3′). Morpholinos for *eNOS* and *prkg1* (for α and β variant) were designed and tested before the experiments and the most efficient sequences were found to be 5′-TTCAGCTTCAATGCTCATACCTGCC-3′ (*prkg1* MO) and 5′-AAAAGCCAAGCACTACTCACCGTTT-3′ (*eNOS* MO), respectively. Morpholinos were injected at the one-cell stage unless otherwise stated. MOs were injected at concentrations: *nNOS* MO 17 ng, *eNOS* MO 34 ng, *notch1* MO 17 ng, *prkg1* MO 51 ng and in parallel appropriate amount of control MO.

### RNA-Seq

Embryos were injected at the one-cell stage using 17 ng of *nNOS*:*eNOS* MO (1:1) mixture and control embryos with identical amount of control MO. Three biological replicates of 10 embryos were collected at stage 26, homogenized in RLT buffer (Qiagen, 74104) and stored at −80°C. Total RNA was isolated using Minikit with standard protocol including DNase treatment (Qiagen, 74104). Quality of total RNA was tested using Experion High sense chip with standard protocol (Biorad, 7007105). RNA samples were sent to EMBL Genomics Core Facility for quantification and library preparation. Libraries were prepared from poly-A selected RNA by Illumina TruSeq RNA Library Preparation Kit v2 (Illumina, RS-122-2001). Libraries were pooled and sequenced using Illumina HiSeq 2500 platform with 50 bp paired-end mode and sequencing depth of approximately 36 million reads per sample.

Data analysis was performed in-house. Ribosomal RNA reads were filtered out by SortMeRNA version 2.0 (Kopylova et al., 2012) and low quality reads and adaptors were filtered out by Trimmomatic version 0.35 for paired end reads (Bolger et al., 2014) using parameters: ILLUMINACLIP:TruSeq3-PE.fa:2:30:10 LEADING:3 TRAILING:3 SLIDINGWINDOW:4:15 MINLEN:36. Reads were than aligned to *Xenopus laevis* genome version 9.1 using STAR version 2.4.2a (Dobin et al., 2013) with default parameters. Count tables were generated by htseq-count using annotation version 1.8.0. Differential expression was analysed using DESeq2 (Love et al., 2014) with default parameters.

### Whole-mount *in situ* hybridization

Whole-mount *in situ* hybridization was performed according to (Sive et al., 2000). Clones of *foxa1* (MXL1736-202771841), *foxi1e* (MXL1736-202771935) and *α-tubulin* (MXL1736-202771973) were purchased from Dharmacon (Lafayette, Colorado, USA). Full-length coding sequences for *eNOS* and *nNOS* were cloned into pBlueScript KS+ vector using primers *eNOS* (forward ATGGGGAGCTCGGCCAGT; reverse TTTCTCTCTCTTCTCCTGTAGG) and *nNOS* (forward ATGGAAGAATATGAGTTCAGCGTT; reverse TTATGAGCAGAAAACCTCATCTGA). The constructs were linearized and transcribed with T7 RNA polymerase (Agilent, 600123-51) to generate antisense RNA probes for *foxi1e*, *foxa1* and *α-tubulin* and T3 RNA polymerase (Agilent, 600111-51) for *eNOS* and *nNOS*. Embryos were fixed in 4% paraformaldehyde (PFA) overnight and incubated in methanol for at least 4 h at −20°C. The proteinase K treatment was removed from original protocol. Samples were imaged on macroscope (Nikon SMZ 1500) and analyzed using ImageJ (NIH, version 1.42q) and Photoshop (Adobe, version 11.0).

### Nitric oxide staining

Embryos were incubated in NO-indicator 4-Amino-5-Methylamino-2′:7′-Difluorofluorescein Diacetate (1:150), (DAF-FM DA – Life Technologies D-23842) together with NO-indicator 5,6-Diaminofluorescein diacetate (1:150) (DAF-2DA – Cayman 85165) for 30 min at room temperature. Embryos were than fixed in 4% PFA for 1 h at room temperature (or overnight at 4°C), counterstained using DAPI (1:1000, Sigma-Aldrich 32670-MG-F) and primary antibodies against α-tubulin (1:1000, Sigma-Aldrich T9026) or Serotonin (5HT) (1:500, Merck Millipore AB938). Samples were imaged using Leica TCS SPE confocal microscope. Images were analyzed using LAS AF Lite (version 2.6.0), ImageJ (NIH) and Photoshop (Adobe).

### Collagens analysis

Paraffin-embedded histological sections were stained using Masson's trichrome staining kit (Sigma-Aldrich, HT15) for the direct visualization of collagens according to manufacturer's instructions.

### Immunofluorescence staining

Immunohistochemistry was performed according to Sive et al. (2000) using primary antibodies against monoclonal α-tubulin (1:1000, Sigma-Aldrich T9026), monoclonal gamma-tubulin (1:1000, Sigma-Aldrich T6557), polyclonal Serotonin (5HT) (1:500, Merck Millipore AB938), Alexa Fluor 488 phalloidin (1:1000, Life Technologies A12379). Alexa 647 goat anti-rabbit (1:250, Life technologies A21244) and Alexa 647 goat anti-mouse (1:500, Life Technologies A21235) were used as secondary antibodies. Samples were imaged using Leica TCS SPE confocal microscope. Images were analyzed using LAS AF Lite, ImageJ (NIH) and Photoshop (Adobe).

### NO donor and inhibitor experiments

NO donor, S-Nitroso-N-acetyl-DL-penicillamine (SNAP, Sigma-Aldrich N3398) was diluted to 100 mM in DMSO. 1 nl of SNAP was injected into oocytes. Embryos were collected at tailbud (stage 30) for immunohistochemistry.

NOS inhibitor 1-(2-Trifluoromethylphenyl) imidazole (TRIM, Sigma-Aldrich T7313) was diluted to 1 M concentration in DMSO and embryos at stage 10, 15, 18 and 20 with manually removed vitelline membrane were incubated in solution of 2 µl of TRIM per 1 ml of 0.1× MBS till stage 26. Embryos were then collected and fixed for immunohistochemistry.

The inhibitor of soluble guanylate cyclase, 1H-[1,2,4]Oxadiazolo[4,3-a]quinoxalin-1-one (ODQ, Sigma-Aldrich O3636) was diluted to 100 mM in DMSO and embryos at stage 10 with manually removed vitelline membrane were incubated in solution of 2 µl of ODQ per 1 ml of 0.1× MBS till stage 26. Embryos were then collected and fixed for immunohistochemistry.

### Collection of embryonic epidermis

Embryos at stage 26 were decapitated and incubated in papain solution (Merck millipore, L2223) for 15 min. Epidermis was manually collected into TRI Reagent for RNA extraction.

## References

[BIO023739C1] Albina J. E., Cui S., Mateo R. B. and Reichner J. S. (1993). Nitric oxide-mediated apoptosis in murine peritoneal macrophages. *J. Immunol.* 150, 5080-5085. 7684418

[BIO023739C2] Alpert M. H., Zhang H., Molinari M., Heitler W. J. and Sillar K. T. (2007). Nitric oxide modulation of the electrically excitable skin of Xenopus laevis frog tadpoles. *J. Exp. Biol.* 210, 3910-3918. 10.1242/jeb.009662 17981858

[BIO023739C3] Arnold W. P., Mittal C. K., Katsuki S. and Murad F. (1977). Nitric oxide activates guanylate cyclase and increases guanosine 3′:5′-cyclic monophosphate levels in various tissue preparations. *Proc. Natl. Acad. Sci. USA* 74, 3203-3207. 10.1073/pnas.74.8.3203 20623 PMC431498

[BIO023739C4] Billett F. S. and Gould R. P. (1971). Fine structural changes in the differentiating epidermis of Xenopus laevis embryos. *J. Anat.* 108, 465-480. 5575314 PMC1234183

[BIO023739C5] Bogdan C. (2001). Nitric oxide and the regulation of gene expression. *Trends Cell Biol.* 11, 66-75. 10.1016/S0962-8924(00)01900-0 11166214

[BIO023739C6] Bolger A. M., Lohse M. and Usadel B. (2014). Trimmomatic: a flexible trimmer for illumina sequence data. *Bioinformatics* 30, 2114-2120. 10.1093/bioinformatics/btu170 24695404 PMC4103590

[BIO023739C7] Bredt D. S., Hwang P. M., Glatt C. E., Lowenstein C., Reed R. R. and Snyder S. H. (1991). Cloned and expressed nitric oxide synthase structurally resembles cytochrome P-450 reductase. *Nature* 351, 714-718. 10.1038/351714a0 1712077

[BIO023739C8] Brunelli E., Perrotta I., Talarico E. and Tripepi S. (2005). Localization of two nitric oxide synthase isoforms, Enos and Inos, in the skin of Triturus Italicus (Amphibia, Urodela) during development. *Comp. Biochem. Physiol. A Mol. Integr. Physiol.* 142, 249-255. 10.1016/j.cbpa.2005.07.004 16139538

[BIO023739C9] Chang C. and Hemmati-Brivanlou A. (1998). Cell fate determination in embryonic ectoderm. *J. Neurobiol.* 36, 128-151. 10.1002/(SICI)1097-4695(199808)36:2<128::AID-NEU3>3.0.CO;2-3 9712300

[BIO023739C10] Chang H.-R., Tsao D.-A., Wang S.-R. and Yu H.-S. (2003). Expression of nitric oxide synthases in keratinocytes after Uvb irradiation. *Arch. Dermatol. Res.* 295, 293-296. 10.1007/s00403-003-0433-4 14615895

[BIO023739C11] Cui X., Zhang J., Ma P., Myers D. E., Goldberg I. G., Sittler K. J., Barb J. J., Munson P. J., Cintron Adel P., McCoy J. P. et al. (2005). Cgmp-independent nitric oxide signaling and regulation of the cell cycle. *BMC Genomics* 6, 151 10.1186/1471-2164-6-151 16269079 PMC1312313

[BIO023739C12] Deblandre G. A., Wettstein D. A., Koyano-Nakagawa N. and Kintner C. (1999). A two-step mechanism generates the spacing pattern of the ciliated cells in the skin of Xenopus embryos. *Development* 126, 4715-4728. 10518489 10.1242/dev.126.21.4715

[BIO023739C13] Dobin A., Davis C. A., Schlesinger F., Drenkow J., Zaleski C., Jha S., Batut P., Chaisson M. and Gingeras T. R. (2013). Star: ultrafast universal RNA-seq aligner. *Bioinformatics* 29, 15-21. 10.1093/bioinformatics/bts635 23104886 PMC3530905

[BIO023739C14] Dubaissi E. and Papalopulu N. (2011). Embryonic frog epidermis: a model for the study of cell-cell interactions in the development of mucociliary disease. *Dis. Model. Mech.* 4, 179-192. 10.1242/dmm.006494 21183475 PMC3046089

[BIO023739C15] Dubaissi E., Rousseau K., Lea R., Soto X., Nardeosingh S., Schweickert A., Amaya E., Thornton D. J. and Papalopulu N. (2014). A secretory cell type develops alongside multiciliated cells, ionocytes and goblet cells, and provides a protective, anti-infective function in the frog embryonic mucociliary epidermis. *Development* 141, 1514-1525. 10.1242/dev.102426 24598166 PMC3957375

[BIO023739C16] Fisher G. J., Kang S., Varani J., Bata-Csorgo Z., Wan Y., Datta S. and Voorhees J. J. (2002). Mechanisms of photoaging and chronological skin aging. *Arch. Dermatol.* 138, 1462-1470. 10.1001/archderm.138.11.1462 12437452

[BIO023739C17] Francis S. H., Busch J. L., Corbin J. D. and Sibley D. (2010). Cgmp-dependent protein kinases and Cgmp phosphodiesterases in nitric oxide and Cgmp action. *Pharmacol. Rev.* 62, 525-563. 10.1124/pr.110.002907 20716671 PMC2964902

[BIO023739C18] Gouge R. C., Marshburn P., Gordon B. E., Nunley W. and Huet-Hudson Y. M. (1998). Nitric oxide as a regulator of embryonic development. *Biol. Reprod.* 58, 875-879. 10.1095/biolreprod58.4.875 9546715

[BIO023739C19] Handy R. L. C., Harb H. L., Wallace P., Gaffen Z., Whitehead K. J. and Moore P. K. (1996). Inhibition of nitric oxide synthase by 1-(2-Trifluoromethylphenyl) imidazole (Trim) in vitro: antinociceptive and cardiovascular effects. *Br. J. Pharmacol.* 119, 423-431. 10.1111/j.1476-5381.1996.tb16003.x 8886430 PMC1915846

[BIO023739C20] Hayes J. M., Kim S. K., Abitua P. B., Park T. J., Herrington E. R., Kitayama A., Grow M. W., Ueno N. and Wallingford J. B. (2007). Identification of novel ciliogenesis factors using a new in vivo model for mucociliary epithelial development. *Dev. Biol.* 312, 115-130. 10.1016/j.ydbio.2007.09.031 17961536 PMC2225594

[BIO023739C21] Ichihashi M., Ueda M., Budiyanto A., Bito T., Oka M., Fukunaga M., Tsuru K. and Horikawa T. (2003). Uv-induced skin damage. *Toxicology* 189, 21-39. 10.1016/S0300-483X(03)00150-1 12821280

[BIO023739C22] Jackson C. L., Lucas J. S., Walker W. T., Owen H., Premadeva I. and Lackie P. M. (2015). Neuronal Nos localises to human airway cilia. *Nitric Oxide-Biol. Chem.* 44, 3-7. 10.1016/j.niox.2014.11.003 25460324

[BIO023739C23] Jacox L., Sindelka R., Chen J., Rothman A., Dickinson A. and Sive H. (2014). The extreme anterior domain is an essential craniofacial organizer acting through Kinin-Kallikrein signaling. *Cell Rep.* 8, 596-609. 10.1016/j.celrep.2014.06.026 25043181 PMC4135435

[BIO023739C24] Jain B., Rubinstein I., Robbins R. A., Leise K. L. and Sisson J. H. (1993). Modulation of airway epithelial cell ciliary beat frequency by nitric oxide. *Biochem. Biophys. Res. Commun.* 191, 83-88. 10.1006/bbrc.1993.1187 7680560

[BIO023739C25] Jiao J., Han D., Meng N., Jin S. and Zhang L. (2010). Regulation of tracheal ciliary beat frequency by nitric oxide synthase substrate l-arginine. *ORL J. Otorhinolaryngol. Relat. Spec.* 72, 6-11. 10.1159/000265683 20110742

[BIO023739C26] Knowles R. G. and Moncada S. (1994). Nitric oxide synthases in mammals. *Biochem. J.* 298, 249-258. 10.1042/bj2980249 7510950 PMC1137932

[BIO023739C27] Kopylova E., Noe L. and Touzet H. (2012). Sortmerna: fast and accurate filtering of ribosomal RNAs in metatranscriptomic data. *Bioinformatics* 28, 3211-3217. 10.1093/bioinformatics/bts611 23071270

[BIO023739C28] Love M. I., Huber W. and Anders S. (2014). Moderated estimation of fold change and dispersion for RNA-seq data with Deseq2. *Genome Biol.* 15, 550 10.1186/s13059-014-0550-8 25516281 PMC4302049

[BIO023739C29] Mall M. A. (2008). Role of cilia, mucus, and airway surface liquid in mucociliary dysfunction: lessons from mouse models. *J. Aerosol. Med. Pulm. Drug Deliv.* 21, 13-24. 10.1089/jamp.2007.0659 18518828

[BIO023739C30] Marcet B., Chevalier B., Luxardi G., Coraux C., Zaragosi L.-E., Cibois M., Robbe-Sermesant K., Jolly T., Cardinaud B., Moreilhon C. et al. (2011). Control of vertebrate multiciliogenesis by Mir-449 through direct repression of the Delta/Notch Pathway (Vol 13, Pg 693, 2011). *Nat. Cell Biol.* 13, 1280-1280 10.1038/ncb2358 21602795

[BIO023739C31] Marnellos G., Deblandre G. A., Mjolsness E. and Kintner C. (2000). Delta-Notch lateral inhibitory patterning in the emergence of ciliated cells in xenopus: experimental observations and a gene network model. *Pac. Symp. Biocomput.* 5, 326-337. 10.1142/9789814447331_0031 10902181

[BIO023739C32] Marshall W. F. and Kintner C. (2008). Cilia orientation and the fluid mechanics of development. *Curr. Opin. Cell Biol.* 20, 48-52. 10.1016/j.ceb.2007.11.009 18194854 PMC2720100

[BIO023739C33] Mitchell B., Jacobs R., Li J., Chien S. and Kintner C. (2007). A positive feedback mechanism governs the polarity and motion of motile cilia. *Nature* 447, 97-101. 10.1038/nature05771 17450123

[BIO023739C34] Nagata S., Nakanishi M., Nanba R. and Fujita N. (2003). Developmental expression of Xeel, a novel molecule of the Xenopus oocyte cortical granule lectin family. *Dev. Genes Evol.* 213, 368-370. 10.1007/s00427-003-0341-9 12802587

[BIO023739C35] Nieuwkoop P. D. and Faber J. (1994). *Normal Table of Xenopus Laevis (Daudin)*. New York: Garland Publishing Inc.

[BIO023739C36] Noiret M., Mottier S., Angrand G., Gautier-Courteille C., Lerivray H., Viet J., Paillard L., Mereau A., Hardy S. and Audic Y. (2016). Ptbp1 and Exosc9 knockdowns trigger skin stability defects through different pathways. *Dev. Biol.* 409, 489-501. 10.1016/j.ydbio.2015.11.002 26546114

[BIO023739C37] Oberprieler N. G., Roberts W., Riba R., Graham A. M., Homer-Vanniasinkam S. and Naseem K. M. (2007). Cgmp-independent inhibition of integrin Alphaiibbeta3-mediated platelet adhesion and outside-in signalling by nitric oxide. *FEBS Lett.* 581, 1529-1534. 10.1016/j.febslet.2007.02.072 17376438

[BIO023739C38] Palmer R. M. J., Ferrige A. G. and Moncada S. (1987). Nitric oxide release accounts for the biological activity of endothelium-derived relaxing factor. *Nature* 327, 524-526. 10.1038/327524a0 3495737

[BIO023739C39] Peus D., Vasa R. A., Meves A., Pott M., Beyerle A., Squillace K. and Pittelkow M. R. (1998). H2o2 is an important mediator of Uvb-induced Egf-receptor phosphorylation in cultured keratinocytes. *J. Invest. Dermatol.* 110, 966-971. 10.1046/j.1523-1747.1998.00210.x 9620307

[BIO023739C40] Pfeilschifter J., Eberhardt W. and Beck K.-F. (2001). Regulation of gene expression by nitric oxide. *Pflugers Arch.* 442, 479-486. 10.1007/s004240100586 11510878

[BIO023739C41] Pilz R. B., Suhasini M., Idriss S., Meinkoth J. L. and Boss G. R. (1995). Nitric oxide and Cgmp analogs activate transcription from Ap-1-responsive promoters in mammalian cells. *FASEB J.* 9, 552-558. 7737465 10.1096/fasebj.9.7.7737465

[BIO023739C42] Quigley I. K., Stubbs J. L. and Kintner C. (2011). Specification of ion transport cells in the Xenopus larval skin. *Development* 138, 705-714. 10.1242/dev.055699 21266406 PMC3026415

[BIO023739C43] Shimizu Y., Sakai M., Umemura Y. and Ueda H. (1997). Immunohistochemical localization of nitric oxide synthase in normal human skin: expression of endothelial-type and inducible-type nitric oxide synthase in keratinocytes. *J. Dermatol.* 24, 80-87. 10.1111/j.1346-8138.1997.tb02748.x 9065701

[BIO023739C44] Sive H. L., Grainger R. M. and Harland R. M. (2000). *Early Development of Xenopus Laevis: A Laboratory Manual*. New York: Cold Spring Harbor Laboratory Press.

[BIO023739C45] Stubbs J. L., Davidson L., Keller R. and Kintner C. (2006). Radial intercalation of ciliated cells during xenopus skin development. *Development* 133, 2507-2515. 10.1242/dev.02417 16728476

[BIO023739C46] Tapia-Limonchi R., Cahuana G. M., Caballano-Infantes E., Salguero-Aranda C., Beltran-Povea A., Hitos A. B., Hmadcha A., Martin F., Soria B., Bedoya F. J. et al. (2016). Nitric oxide prevents mouse embryonic stem cell differentiation through regulation of gene expression, cell signaling, and control of cell proliferation. *J. Cell. Biochem.* 117, 2078-2088. 10.1002/jcb.25513 26853909

[BIO023739C47] Tsao P.-N., Vasconcelos M., Izvolsky K. I., Qian J., Lu J. and Cardoso W. V. (2009). Notch signaling controls the balance of ciliated and secretory cell fates in developing airways. *Development* 136, 2297-2307. 10.1242/dev.034884 19502490 PMC2729343

[BIO023739C48] Walentek P., Bogusch S., Thumberger T., Vick P., Dubaissi E., Beyer T., Blum M. and Schweickert A. (2014). A novel serotonin-secreting cell type regulates ciliary motility in the mucociliary epidermis of Xenopus tadpoles. *Development* 141, 1526-1533. 10.1242/dev.102343 24598162

[BIO023739C49] Walentek P., Beyer T., Hagenlocher C., Müller C., Feistel K., Schweickert A., Harland R. M. and Blum M. (2015). Atp4a is required for development and function of the Xenopus mucociliary epidermis - a potential model to study proton pump inhibitor-associated pneumonia. *Dev. Biol.* 408, 292-304. 10.1016/j.ydbio.2015.03.013 25848696 PMC4592800

[BIO023739C50] Wang R., Ghahary A., Shen Y. J., Scott P. G. and Tredget E. E. (1996). Human dermal fibroblasts produce nitric oxide and express both constitutive and inducible nitric oxide synthase isoforms. *J. Invest. Dermatol.* 106, 419-427. 10.1111/1523-1747.ep12343428 8648170

[BIO023739C51] West A. R., Galloway M. P. and Grace A. A. (2002). Regulation of striatal dopamine neurotransmission by nitric oxide: effector pathways and signaling mechanisms. *Synapse* 44, 227-245. 10.1002/syn.10076 11984858

[BIO023739C52] Wettstein D. A., Turner D. L. and Kintner C. (1997). The Xenopus homolog of Drosophila suppressor of hairless mediates notch signaling during primary neurogenesis. *Development* 124, 693-702. 9043084 10.1242/dev.124.3.693

[BIO023739C53] Wildling S. and Kerschbaum H. H. (2007). Nitric oxide decreases ammonium release in tadpoles of the clawed frog, Xenopus laevis, Daudin. *J. Comp. Physiol. B* 177, 401-411. 10.1007/s00360-006-0139-y 17211666

[BIO023739C54] Xu J., Kim G.-M., Chen S., Yan P., Ahmed S. H., Ku G., Beckman J. S., Xu X. M. and Hsu C. Y. (2001). Inos and nitrotyrosine expression after spinal cord injury. *J. Neurotrauma* 18, 523-532. 10.1089/089771501300227323 11393255

